# Parkinson Disease: PD Gene and Oxidative Stress

**DOI:** 10.1289/ehp.114-a96a

**Published:** 2006-02

**Authors:** Melissa Lee Phillips

A gene linked to familial Parkinson disease (PD) may protect neurons from oxidative damage, according to two independent studies in the fruit fly *Drosophila*. Flies lacking the *DJ-1* gene showed selective sensitivity to widely used agricultural toxicants that kill neurons mainly through oxidative stress. The studies, published 6 September 2005 in *Current Biology*, suggest that normally “*DJ-1* has a neuroprotective role against different oxidative stimuli,” says Darren Moore, an instructor in neurology at the Johns Hopkins University School of Medicine who has studied the gene. However, if *DJ-1* stops working—because of either an inherited mutation or toxicant exposure—oxidative stress may wreak havoc on the brain, killing dopamine-producing neurons.

Experiments in cultured cells and in knockout mice have hinted that *DJ-1* mutations may sensitize cells to the harmful effects of oxidative stress. This type of oxidative damage happens when unstable oxygen molecules react with certain components inside cells in a manner similar to the process that converts iron to rust. Many environmental insults—including exposure to certain agricultural chemicals—can generate these unstable oxygen molecules.

To pin down how *DJ-1* interacts with such oxidative stress agents, Nancy Bonini, a professor of biology at the University of Pennsylvania, Philadelphia, and her colleagues first identified *DJ-1* in *Drosophila*, which they found exists in two forms: *DJ-1*α (expressed primarily in the testes) and *DJ-1*β (expressed everywhere). They then created a line of *Drosophila* mutants completely lacking both forms.

The flies with no *DJ-1* had normal life spans and showed no neuronal degeneration. However, when Bonini and her colleagues exposed flies to the herbicide paraquat, *DJ-1* mutants died much sooner than normal flies. The mutants also showed marked sensitivity to the insecticide rotenone and to hydrogen peroxide—both agents that promote oxidative stress. These results suggest that *DJ-1* normally protects against oxidative stress and that its inactivation may leave neurons susceptible to oxidative damage. The team also found that exposure to paraquat led to biochemical modification of the DJ-1β protein, a change Bonini says may somehow influence the ability of *DJ-1* to protect neurons from oxidative damage.

In the other paper, Kyung-Tai Min, an investigator at the National Institute of Neurological Disorders and Stroke, and his colleagues examined a different type of *Drosophila DJ-1* mutant. They disrupted the function of *DJ-1*β by inserting a mutation into the middle of the gene. Surprisingly, Min says, they found that dopamin-ergic neurons in these mutants survived longer into old age than did neurons of normal flies. They also found that their *DJ-1*β mutants were much more resistant to paraquat insult than were normal flies.

Further examination revealed that these flies had elevated *DJ-1*α expression—the loss of *DJ-1*β somehow encouraged a compensatory upregulation of *DJ-1*α, which the authors believe protected the fly from paraquat-induced oxidative damage. When they treated the same flies with hydrogen peroxide, however, they found that the *DJ-1*β mutants were extremely susceptible to early death. Min says this suggests that *DJ-1*α and *DJ-1*β may normally protect cells against different types of agents that promote oxidative stress.

Neither of the mutant *DJ-1* fly strains will probably make an ideal model for PD, according to Moore, because the flies don’t suffer from the neurodegeneration seen in humans with *DJ-1* mutations. Nevertheless, says Bonini, studies of *DJ-1* in *Drosophila* will provide greater understanding of fundamental activities of the gene, helping to elucidate how its function may be critical in PD.

